# Wet catalytic oxidation upcycling semi-dry flue gas desulfurization ash to sustainable gypsum

**DOI:** 10.1016/j.isci.2025.113476

**Published:** 2025-09-07

**Authors:** Hao-Qin Xiong, Jia-Zhuo Qu, Zhe-Xi Luan, Xiao-Long Sun, Xiang-Yang Mei

**Affiliations:** 1 Yunnan Key Laboratory of Plateau Wetland Conservation, Restoration and Ecological Services, College of Ecology and Environment, Southwest Forestry University, Kunming 650224, P.R. China; 2 Yunnan Academy of Ecological and Environmental Sciences, Kunming 650034, P.R. China; 3 Yunnan Appraisal Center for Ecological and Environmental Engineering, Kunming 650228, P.R. China

**Keywords:** Catalysis, Environmental engineering, Chemical processing, Nanomaterials

## Abstract

Semi-dry flue gas desulfurization ash (SFGDA), rich in calcium sulfite (CaSO_3_) and highly alkaline, poses challenges for direct reuse due to its instability and slow oxidation. This review synthesizes recent advances in wet catalytic oxidation (WCO) that transform CaSO_3_ into construction-grade calcium sulfate (CaSO_4_). We frame homogeneous and heterogeneous radical-chain routes and lattice-defect activation, underscoring how Mn/Co/Fe redox cycles and oxygen-vacancy engineering accelerate conversion. Performance comparisons span conventional salts, metal-organic frameworks (MOFs), perovskites, and nanostructured catalysts, with Co-MOFs achieving 96% oxidation in 3 h and MnTiO_3_ lowering activation energy by 40%. Process optimizations ultrasound assisted nanobubbles, micro-scale mass-transfer intensification, and pH control at 5–6 address diffusional and alkaline limitations, while layered double hydroxides and anti-scaling surfaces mitigate chloride poisoning and fouling. By merging mechanistic insights with technological progress, this work maps out a sustainable pathway for SFGDA management.

## Graphical abstract

**Figure:**
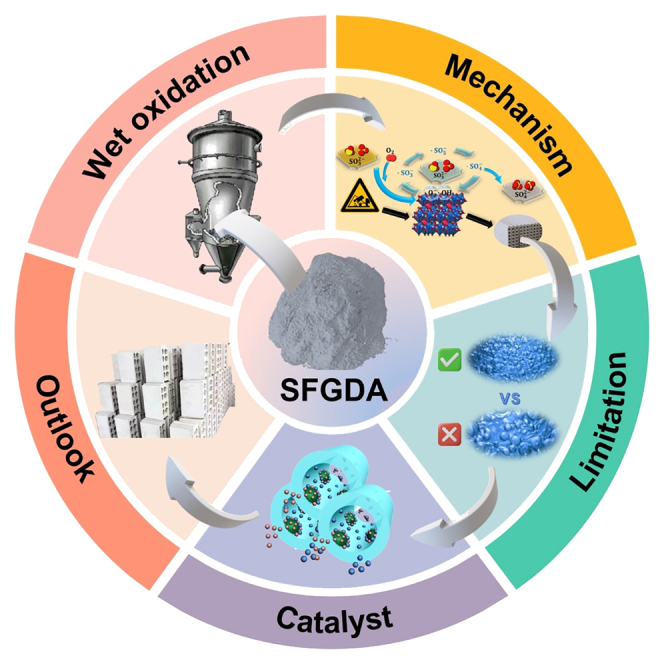

## Introduction

Sulfur dioxide (SO_2_) is a prominent atmospheric pollutant with the potential to initiate secondary environmental issues, such as acid rain and smog, thereby posing significant threats to ecosystems and human health.^1^^,^^2^^,^^3^ To alleviate the adverse environmental effects of SO_2_ emissions, numerous flue gas desulfurization (FGD) technologies, encompassing wet, dry, and semi-dry methods, have been extensively adopted in industrial sectors for the efficient capture and fixation of sulfur dioxide from flue gases.^4^^,^^5^^,^^6^

**Figure 1:**
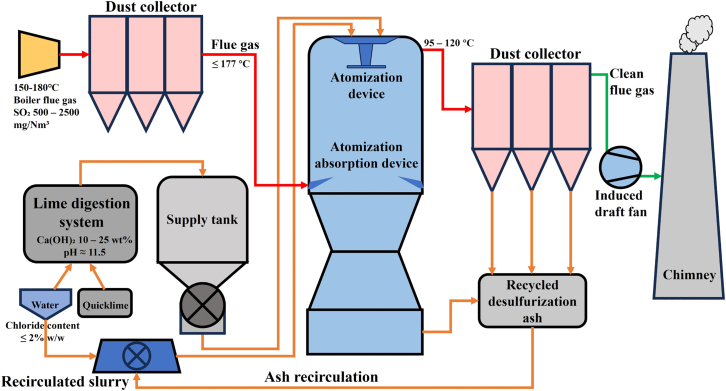
Semi-dry flue gas desulfurization process

As illustrated in Figure 1, the semi-dry calcium-based desulfurization (SFGD) process comprises three key stages: slurry preparation, spray absorption, and particulate collection. First, quicklime (CaO) reacts with water to produce a calcium hydroxide (Ca(OH)_2_) slurry. The slurry is then delivered to a spray absorber, where it is atomized into micron-sized droplets that instantly contact flue gas at 150°C–180°C. Within seconds, the droplets dry; SO_2_ is absorbed and reacts with Ca(OH)_2_ to form CaSO_3_ as the main product, with a minor fraction further oxidized to CaSO_4_. The resulting dry powder is captured by an electrostatic precipitator, yielding SFGDA. Because the droplets dry rapidly, the disappearance of the water film deprives O_2_ and SO_3_^2−^ of a diffusion medium, so the newly formed CaSO_3_ becomes encapsulated and resists further oxidation. Moreover, excess lime is dosed to maintain desulfurization efficiency, leaving residual unreacted Ca(OH)_2_ that further lowers the oxidation extent. Consequently, the final ash is dominated by CaSO_3_ and Ca(OH)_2_, with relatively low CaSO_4_ content.^7^^,^^8^

Wet flue-gas desulfurization (WFGD) consists of three main subsystems: slurry preparation, the absorber tower, and by-product handling. Inside the absorber, a slurry of limestone (CaCO_3_) or calcium hydroxide contacts the upward-flowing flue gas counter-currently. SO_2_ is absorbed and converted to sulfites. With CaCO_3_ as the sorbent, a forced-oxidation step converts sulfites to stable gypsum (CaSO_4_·2H_2_O).^4^^,^^9^ Using CaO, however, results in low calcium utilization, high residual Ca(OH)_2_ and CaSO_3_ contents, and poor oxidizability.

Dry flue-gas desulfurization (DFGD) removes SO_2_ by thoroughly mixing the flue gas with a dry powder sorbent. The process is a gas–solid reaction with a slow rate, which limits desulfurization efficiency and calcium utilization. When quicklime is used as the sorbent, the residue contains considerable CaSO_3_ and unreacted CaO, hindering the utilization of the gypsum by-product.^6^^,^^10^

Owing to the limitations of the SFGD process, CaSO_3_ in SFGDA is difficult to undergo further oxidation. However, the high CaSO_3_ content, which is chemically unstable and has low reactivity, poses significant challenges to its direct application. Consequently, most SFGDA is currently disposed of via landfilling, a practice that consumes considerable land resources and poses potential environmental hazards.^11^^,^^12^ Thus, the efficient stabilization and conversion of CaSO_3_ in SFGDA—specifically through oxidation to the more stable CaSO_4_—is crucial for realizing its sustainable reuse.

**Figure 2:**
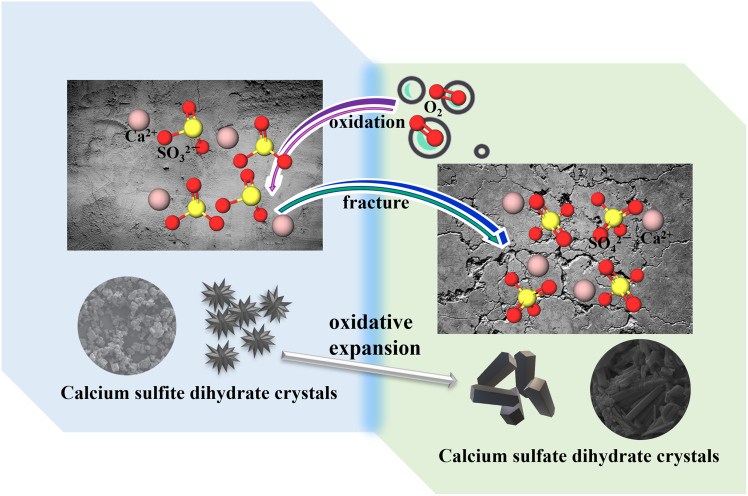
Cracking of gypsum caused by the use of semi-dry flue gas desulfurization ash as building gypsum

SFGDA commonly occurs as a fine powder with a broad particle size distribution, a porous structure, and a high specific surface area. Its principal components include CaSO_3_, CaSO_4_, Ca(OH)_2_, CaCO_3_, and CaCl_2_.^13^^,^^14^ The presence of CaO and Ca(OH)_2_ contributes to the strong alkalinity of SFGDA (pH ≈ 12) and its high water absorption capacity, along with considerable hydration reactivity.^15^ Nevertheless, upon exposure to air, the CaSO_3_ component in SFGDA gradually oxidizes and expands, leading to cracking and degradation in the strength of gypsum-based products, as depicted in Figure 2.^16^ Therefore, the development of an efficient, environmentally friendly, and controllable oxidation pathway for converting CaSO_3_ in SFGDA to the stable CaSO_4_ is vital not only for mitigating the environmental impact of SFGDA accumulation but also for facilitating its large-scale adoption in construction and related fields.

Currently, the reoxidation of CaSO_3_ in SFGDA is predominantly achieved through dry and wet oxidation processes, with microbial oxidation gaining traction as an approach under active research.^13^^,^^17^^,^^18^ As shown in Table 1, the dry oxidation of CaSO_3_ is simple and straightforward, relying on gas-solid reactions alone. However, it is hampered by limitations such as limited contact area between phases, slow oxidation rates, and high energy consumption, necessitating elevated temperature and pressure. These factors collectively diminish its economic feasibility.^14^^,^^19^^,^^20^ While the microbial oxidation of CaSO_3_ offers favorable operating conditions and minimal reagent requirements, its slow reaction pace, complex nutritional demands of microbial strains, and challenges in process control restrict its potential for industrial-scale applications.^18^ Conversely, wet oxidation has garnered significant attention for its cost-effectiveness and efficiency. In particular, WCO has become a focal point of research due to its rapid reaction kinetics, high oxidation efficiency, and ease of operation. This method stands out as one of the most promising to date.^21^^,^^22^ Compared to conventional thermal oxidation or microbial methods, WCO offers several distinct advantages, including high oxidation efficiency and the production of stable end-products, primarily CaSO_4_·2H_2_O or CaSO_4_, which are well-crystallized and structurally sound. These products can be used directly as gypsum in construction or agriculture, mitigating concerns such as swelling and cracking.^23^ The process also provides robust control over reaction conditions, allowing for the precise adjustment of operational parameters to manage reaction kinetics and product crystal morphology, aligning with the requirements of SFGD processes.^24^ Furthermore, it presents minimal environmental risk, as it does not emit secondary gaseous pollutants, and the residual liquid can be recycled, enhancing the process’s safety and sustainability over alternative treatments.^25^ Consequently, WCO offers an efficient and environmentally friendly route for the high-value utilization of SFGDA, supporting broader objectives such as “waste-to-resource” conversion and synergistic carbon reduction. It represents a crucial strategy for the green disposal of flue gas desulfurization by-products.

**Table 1:** Comparison of three CaSO_3_-oxidation routes

Technology	CaSO_3_ conversion rate	Reaction temperature	Advantages	Limitations	Product utilization potential	Industrial application scenario
Dry thermal oxidation	80–90% conversion within 2 h	High (400°C–550°C)	High efficiency; simple system; gypsum-ready product	Limited contact; energy-intensive; Cl^−^ sensitive	High suitability as gypsum/cement retarder	Semi-industrial trial; no full-scale plant
Wet catalytic oxidation	75–80% conversion within 2 h	Low (25°C–60°C)	Ambient conditions; low risk; continuous operation	Catalyst cost and liquid removal required	High-purity CaSO_4_·2H_2_O usable in boards/cement	Adaptable to semi-dry FGD; scalable
Biological oxidation	Over 95% conversion within 10 days	Very low (20°C–25°C)	Low-cost; low additive demand; large-scale feasible	Long period and slow rate; complex bio-maintenance; land-demanding	Applicable in gypsum board, cement, and soil remediation	Lab-scale only; not yet industrialized

Note: Data (refs.^14^^,^^17^^,^^18^^,^^19^^,^^20^^,^^21^^,^^22^) are illustrative and for qualitative comparison only.

Recent years have seen substantial advancements in the utilization of flue gas desulfurization gypsum, notably through WCO strategies. On one hand, the elucidation of free radical chain reactions and catalyst site-occupancy mechanisms has bolstered the theoretical foundation for CaSO_3_ wet catalytic oxidation.^26^^,^^27^ On the other hand, improvements in reaction conditions, such as micro/nanobubble-assisted mass transfer, external additive regulation, and the development of alkaline environment-appropriate catalysts, have introduced innovative methods to enhance CaSO_3_ oxidation efficiency. Moreover, the development of high-performance catalysts, including MOFs,^28^^,^^29^ perovskite structures,^30^ and nanocomposite materials,^31^^,^^32^ has overcome limitations of traditional systems and significantly enhanced both radical generation efficiency and reaction selectivity. Summarizing the recent progress in WCO of SFGDA is highly significant for fostering the high-value utilization of this industrial solid waste type, as well as facilitating pollutant reduction and integration into building material applications. Although research has explored the properties and treatment methods of SFGDA,^5^^,^^7^ a comprehensive review specifically addressing its WCO mechanisms and catalyst applications is currently lacking and is deemed urgent.

In this review, we offer a comprehensive overview of the WCO mechanisms and reaction pathways of CaSO_3_ within semi-dry flue gas desulfurization ash (SFGDA). We focus on the mechanistic roles of key influential factors and provide an in-depth comparison of mainstream catalysts based on their catalytic activity, stability, and adaptability. Building on this, we discuss current challenges and future directions for applying CaSO_3_ catalytic wet oxidation to the material utilization of SFGDA, aiming to offer insights for both fundamental research and engineering applications in the field. To facilitate the rapid comprehension of these relationships, Table S1 presents a three-dimensional matrix. Each catalyst type is tagged with its investigated mechanisms (lattice-defect or radical-chain pathways) and limiting factors (mass transfer, pH, Cl^−^, additives, and so forth), thereby visualizing research hotspots and gaps. It should be noted that this review is specifically centered on SFGDA and may have limited applicability to desulfurization ash from dry or wet FGD processes.

## Mechanism of catalytic oxidation

**Figure 3:**
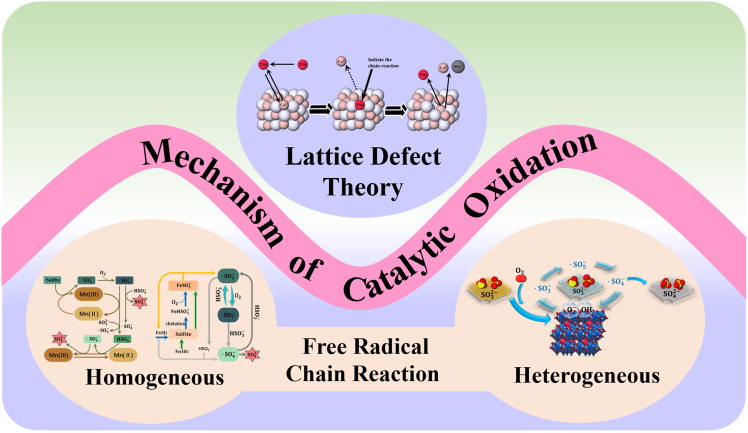
Catalytic oxidation mechanism relationship diagram

The current mechanistic understanding of CaSO_3_ catalytic oxidation is rooted in two dominant hypotheses: the free radical chain reaction mechanism and the catalyst site-occupancy hypothesis.^26^^,^^27^ The free radical chain mechanism highlights the significance of radical initiation, propagation, and termination in driving the oxidation process.^33^ In contrast, the site-occupancy hypothesis posits that the adsorption of catalysts at distinct sites on the CaSO_3_ surface modifies the oxidation pathway.^34^ The specific catalytic oxidation mechanism is shown in Figure 3.

### Lattice defect theory

**Figure 4:**
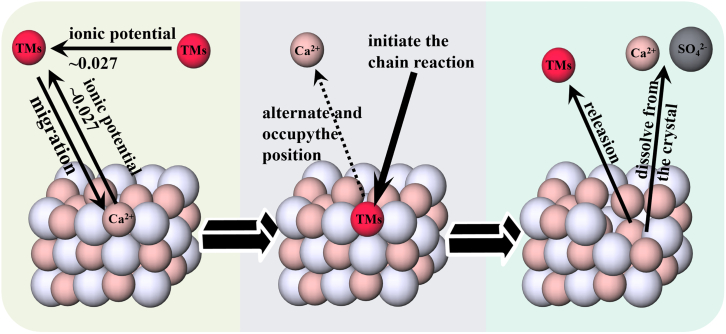
Schematic diagram of the lattice defect theory

During the WCO of CaSO_3_, alongside conventional liquid-phase oxidation pathways, research indicates that catalysts can directly engage with solid-phase CaSO_3_ crystals, thereby significantly boosting their oxidation efficiency. The selective catalytic activity of transition metals (TMs) toward sulfites, particularly in solid-state systems, is frequently attributed to their site-occupancy properties within the crystal structure (as depicted in Figure 4).^35^^,^^36^ Fundamentally, lattice defect theory proposes that the surfaces of CaSO_3_ crystals are riddled with point defects, such as vacancies, interstitial atoms, and dislocated ions, which are thought to be pivotal active sites for catalytic oxidation.^37^^,^^38^^,^^39^

At the microscopic level, the irregular surface structure of CaSO_3_ causes alterations in local electron density and coordination environments, enhancing its capacity to adsorb external ions. Given the similarity between Ca^2+^ and TM ions (e.g., Co^2+^, Fe^3+^, Mn^2+^) in terms of ionic radius, charge state, and outer electron configuration, TM ions can selectively fill Ca2+ defect sites on the surface lattice through a site-occupancy process.^40^ This mechanism not only ensures the stable binding of TM ions but may also promote the formation of highly active intermediate sulfite complexes. These intermediates, containing redox-flexible TM elements (e.g., Mn^2+^ ⇌ Mn^3+^), possess robust redox potential and readily react with molecular oxygen in the system, SO_3_^2−^ to sSO_4_^2−^, thereby completing the catalytic cycle. Following this, the TM ions disengage from the reaction complex and migrate to fresh defect sites, resuming their catalytic role. Concurrently, the CaSO_3_ surface layer gradually peels away, continually revealing fresh surfaces that supply a steady stream of active sites for ongoing reactions.^40^ Evidence suggests that TM ions such as Co, Fe, and Mn display superior catalytic activity and selectivity in these processes.^41^

The site-occupancy catalytic mechanism underscores the indispensable role of lattice defects in solid-liquid interfacial oxidation, providing a theoretical basis for the effective transformation of sulfites in high-concentration, partially dissolved FGD ash slurry systems. This theory is of profound importance for comprehending the synergistic catalytic oxidation dynamics of solid residues in SFGD processes. It also presents new viewpoints and mechanistic insights for formulating catalytic models tailored to complex slurry-phase systems on an industrial scale.

### Free radical chain reaction

#### Homogeneous catalytic system

**Figure 5:**
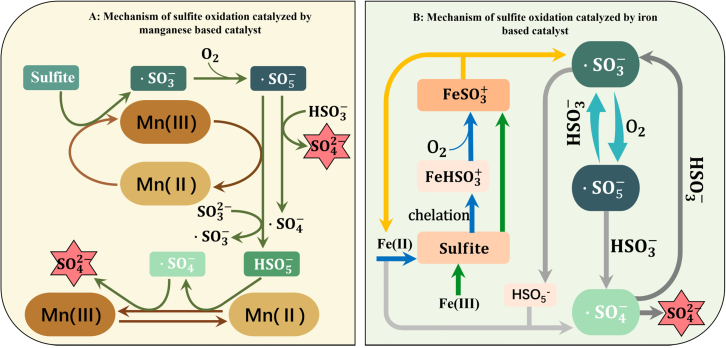
Mechanism of free radical chain reaction in homogeneous catalytic systems (A) Mechanism of sulfite oxidation catalyzed by manganese based catalyst. (B) Mechanism of sulfite oxidation catalyzed by iron based catalyst.

In homogeneous catalytic systems, transition metal salts are among the most commonly used catalysts. Among them, manganese-based and iron-based catalysts have been most extensively studied for sulfite oxidation. Their catalytic processes are typically accompanied by free radical chain reactions,^42^^,^^43^^,^^44^ as illustrated in Figure 5.

The oxidation of CaSO_3_ catalyzed by Mn^2+^ typically involves a free radical chain reaction mechanism, beginning with a chain initiation step, in which (SO_3_^2─^ are activated by Mn^2+^ to form sulfite radicals (SO_3_·−) and reduced Mn species (Me^(n−1)+^); followed by a chain propagation step, where SO_3_·− reacts with O_2_ to generate peroxysulfate radicals (SO_5_·−); SO_5_·− further reacts with bisulfite (HSO_3_^─^) and SO_3_^2─^ to produce peroxymonosulfate (HSO_5_^─^), sulfate ions (SO_4_^2─^), sulfate radicals (SO_4_·−), and additional SO_3_·−; in the termination step, HSO_5_^−^ is catalytically decomposed by Mn species to generate OH^−^ and SO_4_·− radicals, which subsequently react with Me^(n−1)+^ to produce SO_4_^2─^ and regenerate Mn^2+^. Additionally, SO_4_·− may also undergo self-decomposition to yield SO_4_^2─^.^42^

In the Fe^2+^-catalyzed CaSO_3_ oxidation system, Fe^2+^ first forms a chelated complex with sulfite to produce FeHSO_3_^+^, which subsequently reacts with O_2_ to generate FeSO_3_^+^. FeSO_3_^+^ then decomposes into Fe^2+^ and SO_3_·−, with Fe^2+^ re-entering the activation and complexation cycle. The SO_3_·− radicals, in the presence of oxygen, generate SO_5_·−, which subsequently react with HSO_3_^−^ to produce SO_4_·−. In addition, SO_5_·− can further react with HSO_3_^−^ to regenerate SO_3_·−, which then reacts again with HSO_3_^−^ to form HSO_5_^─^. In the presence of Fe^2+^, HSO_5_^─^ is catalytically decomposed to generate SO_4_·−, which is ultimately reduced to SO_4_^2─^. Alternatively, SO_4_·− may also undergo self-decomposition to produce SO_4_^2─^. Similar to Fe^2+^, when Fe^3+^ initiates the reaction, the activation of sulfite also occurs via the formation of FeSO_3_^+^ complexes.^43^

Based on the above, the construction of Mn/Fe bimetallic homogeneous catalytic systems offers significant potential for synergistic effects. On one hand, Mn^2+^ exhibits stronger initial activation of SO_3_^2−^ and higher efficiency in initiating radical chain reactions; on the other hand, Fe^2+^ facilitates the sustained generation and stabilization of SO_4_·− radicals, which helps extend radical lifetimes and suppress side reactions. Moreover, a cross-coupled redox cycle between Mn and Fe redox pairs is expected to enhance the overall electron transfer efficiency and radical concentration, thereby increasing both the reaction rate and selectivity. Previous studies have demonstrated that multimetallic catalytic systems exhibit superior radical generation capacity and catalytic stability compared to single-metal systems in various homogeneous oxidation reactions.^45^ For example, in CaSO_3_-activated systems and reactive oxygen chain reaction networks involving transition metals, combinations such as Mn–Fe and Fe–Ce have shown pronounced synergistic effects.^46^^,^^47^ Therefore, introducing multimetallic synergistic mechanisms into CaSO_3_ wet catalytic oxidation systems is expected to improve the conversion efficiency from CaSO_3_ to CaSO_4_, lower the activation energy, and enhance catalyst resistance to deactivation.^48^

Although multi-metal combinations exhibit significant synergistic potential in the catalytic oxidation of CaSO_3_, their homogeneous catalytic mechanisms are far more complex than those of single-metal systems.^45^^,^^49^ This complexity manifests in several aspects. Differences in redox potentials among metal ions can trigger competitive reactions or mutual passivation.^50^^,^^51^ For example, electron transfer between transition-metal ions can alter their positions and pathways within the radical chain. The types and concentrations of radicals often show nonlinear behavior.^52^ Under multi-metal conditions, different ions can initiate multiple radical-generation routes; thus, the conversion rates and stabilities of intermediates such as SO_4_·−, SO_5_·−, and HSO_5_^−^ may exhibit synergistic or antagonistic effects, making the system prone to by-product accumulation or radical-chain termination.^53^ Therefore, these complexities must be fully considered when designing homogeneous multi-metal catalytic systems.

#### Heterogeneous catalytic system

**Figure 6:**
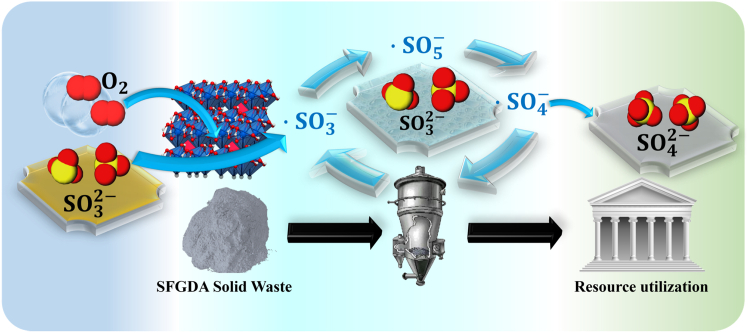
Mechanism of free radical chain reaction in heterogeneous catalytic systems

In heterogeneous catalytic systems, the oxidation mechanism of CaSO_3_ is also interpreted as a radical chain reaction, as illustrated in Figure 6.^54^ The catalytic oxidation of CaSO_3_ occurs on the surface of solid catalysts, where the radical chain reaction is initiated at the active sites of the catalyst, leading to the generation of reactive oxygen species (ROS: superoxide radical (O_2_·−) and hydroxyl radical (·OH).) on the catalyst surface. These species further promote the formation and propagation of sulfur–oxygen radicals (SO_5_·−, SO_4_·−, and SO_3_·−) involved in the radical chain reaction, among which the sulfate radical (SO_4_·−) serves as the key precursor for the formation of sulfate ions (SO_4_^2−^). As shown in the Figure 6, O_2_ molecules are adsorbed onto the catalyst surface and subsequently activated to form reactive oxygen species, including O_2_·− and ·OH.^55^ Subsequently, SO_3_^2−^ ions are also adsorbed onto the catalyst surface, where they react with the reactive oxygen species to form SO_5_·− and SO_4_·− radicals, with SO_4_·− eventually converting into SO_4_^2−^ in the aqueous phase and being released from the catalyst surface.^56^

In summary, research on the catalytic oxidation mechanisms of sulfites has primarily focused on single-component catalysts, while the catalytic rate and overall oxidation efficiency of single catalysts have reached performance limits in many systems, leading to increasing attention on multi-catalyst synergistic systems.^57^ However, the mechanistic understanding of these synergistic systems remains limited due to the increased complexity of multi-catalyst interactions.^58^ In composite catalysts, synergistic interactions between different metal components can significantly enhance catalytic activity, but the precise nature of these interactions remains unclear. In particular, how to accurately characterize the roles and cooperative behaviors of individual metals in bimetallic or multimetallic systems requires further investigation.^59^ Moreover, the mechanisms of electron transfer and interfacial interactions between metals and supports have not been fully elucidated, and how these interactions influence catalytic activity, selectivity, and stability remains a central focus of current research.^60^

### Coupling between reaction environment and mechanism

In catalytic systems, the regulatory effects of reaction conditions on catalytic mechanisms should not be overlooked. Studies have shown that O_2_ availability can influence the reaction pathway by affecting the combination of SO_3_·− with O_2_ to form SO_5_·−, and that insufficient oxygen partial pressure may hinder chain propagation and termination processes, thereby reducing the overall oxidation efficiency of CaSO_3_.^61^ The solution pH also significantly affects the valence state stability and complexation ability of metal ions. The Fe^2+^/Fe^3+^ redox system performs optimally under mildly acidic to near-neutral conditions, while Mn^2+^ remains more stable under alkaline conditions. However, the inherent alkalinity of SFGDA systems (pH > 12) restricts the formation pathways of certain radicals, such as ·OH, highlighting the importance of pH control strategies—such as the addition of buffering agents or weak acids—for modulating reaction activity. The dosage and molar ratio of metals are critical in determining whether synergistic, competitive, or antagonistic interactions occur between metal species. For instance, at Fe: Mn molar ratios of 1:1 to 2:1, some studies have observed enhanced generation rates of SO_4_·− radicals, whereas excessive Fe^3+^ concentrations may scavenge SO_3_·− radicals, thereby reducing the overall reaction efficiency.^62^ Therefore, constructing an efficient and stable multimetal homogeneous catalytic system requires not only the rational design and optimization of catalyst composition, but also the careful tuning of reaction parameters and a deep understanding of the limiting factors that influence catalytic oxidation, in order to enable the practical design and industrial implementation of SFGDA catalytic oxidation.

## Limiting factors affecting catalytic oxidation

**Figure 7:**
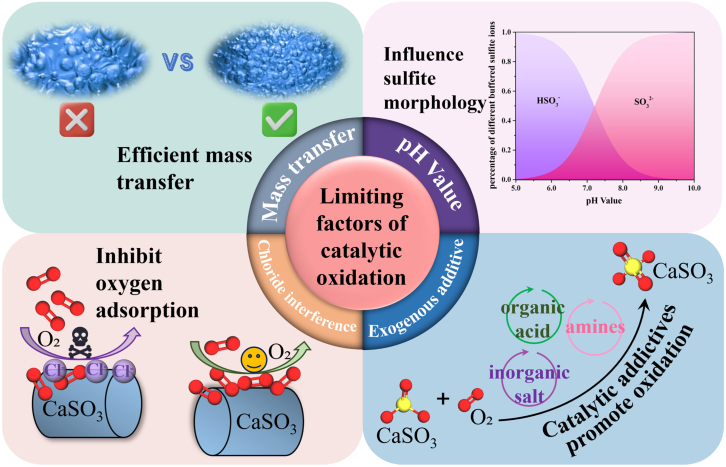
Restrictive factors affecting catalytic oxidation

The WCO of CaSO_3_ is constrained by a range of physical and chemical factors, which collectively determine the feasibility of the catalytic reaction, the efficiency of radical generation, and the overall cost-effectiveness of the process. As shown in Figure 7, key barriers to the efficient catalytic oxidation of SFGDA include mass transfer limitations, pH regulation, Cl^−^ interference, and the effects of external additives.^63^^,^^64^^,^^65^ This section systematically reviews the reaction mechanisms and optimization strategies associated with these factors.

### Mass transfer

In the WCO of CaSO_3_, mass transfer bottlenecks arise at both the gas–liquid interface (O_2_ dissolution into the liquid phase) and the liquid–solid interface (CaSO_3_ dissolution and subsequent reaction), which significantly impact the overall oxidation process.^66^ Factors such as the low dissolution rate of O_2_, poor dispersion of CaSO_3_ particles, and oversized gas bubbles can all limit effective contact between reactants, thereby inhibiting the catalyst’s ability to initiate and sustain the free radical chain reaction.^67^

To overcome these limitations, various technological strategies have been proposed to enhance mass transfer efficiency in gas–liquid–solid three-phase systems.^68^ Nanobubbles (diameter <200 nm), characterized by high specific surface area, strong stability, and slow collapse rates, can continuously release O_2_ into the liquid phase, significantly enhancing dissolved oxygen concentration and radical lifetime. Studies have reported that under nanobubble conditions, the gas–liquid mass transfer coefficient can reach (5.0–8.2) × 10^−3^ s^−1^, which is 20–50 times higher than that of conventional bubbling systems.^69^ This technology has shown great potential in organic pollutant oxidation systems and is considered feasible for application in SFGDA oxidation processes. In addition, ultrasound-assisted techniques utilize cavitation effects to generate high-energy microjets, which greatly enhance bubble fragmentation and liquid turbulence, improving the uniform distribution of O_2_ in the liquid phase. In ozonation systems, cavitation-enhanced ultrasonication has been reported to increase the reaction rate constant by more than 45 times, demonstrating considerable potential for oxidation process intensification.^70^ The introduction of this approach into SFGDA systems could further mitigate the inefficiencies in radical generation caused by delayed O_2_ transport during gas–liquid mass transfer. Microreactors, as the new process intensification devices, utilize microscale channel designs to increase the gas–liquid interfacial area and gas residence time, thereby achieving orders-of-magnitude enhancement in volumetric mass transfer coefficients.^71^ The micro-interface intensified reactor (MIR) developed by Qian et al. is capable of reducing the average bubble diameter to below 10 μm, resulting in a 30- to 60-fold improvement in mass transfer efficiency compared to conventional reactors.^71^ This reactor offers advantages such as modularity and high controllability, providing a scalable pathway for the industrial implementation of the CaSO_3_ oxidation process.^72^^,^^73^

From the perspective of mass transfer limitations in the catalytic oxidation of SFGDA, intensification technologies such as nanobubbles, ultrasonics, and microreactors have demonstrated strong mass transfer enhancement in experimental systems and exhibit promising potential for broader application in SFGDA oxidation processes. Future industrial studies may focus on the synergistic integration of these techniques with catalysts and additives, aiming to develop more efficient and energy-saving three-phase reaction platforms.

### pH value

The WCO of CaSO_3_ is highly sensitive to pH conditions, as pH not only governs the speciation among SO_3_^2−^, HSO_3_^−^, and H_2_SO_3_, but also significantly regulates the types and rates of radical generation as well as the stability of catalytically active sites.^74^ Studies have shown that at a pH range of 5–6, the catalytic oxidation rate of CaSO_3_ reaches its maximum, with a SO_4_^2−^ formation rate of approximately 0.092 mmol·L^−1^·min^−1^; whereas under alkaline conditions (pH > 10), the rate sharply declines to 0.011 mmol·L^−1^·min^−1^, representing a decrease of over 88%.^72^ This trend is theoretically attributed to the role of H^+^ ions in acidic conditions, which facilitate SO_3_^2−^ activation and promote radical chain propagation, yet in industrial practice, maintaining low pH requires large quantities of organic or inorganic acids to adjust the system, which poses practical challenges such as high corrosivity, increased reagent consumption, and operational complexity.^75^

To address the challenge of low CaSO_3_ oxidation efficiency under alkaline conditions, various adaptive enhancement strategies have been proposed. The feasibility of these approaches has been validated at the experimental level. Lian et al. demonstrated that ozone (O_3_)-induced radical oxidation pathways can still yield high radical production rates at pH = 9, with a SO_4_^2−^ formation rate of up to 0.036 mmol·L^−1^·s^−1^, making it a promising intensification pathway without the need to modify the system’s initial pH.^74^ In addition, electrochemical oxidation methods can generate strong oxidants such as ·OH and O_3_ at the anode or cathode without requiring external H^+^ input, and maintain high oxidation capacity under pH conditions ranging from 8 to 11.^76^ Furthermore, the development of heterogeneous catalysts suitable for alkaline conditions can enhance SO_3_^2−^ activation by tuning their electronic structures and oxygen adsorption properties, with reported reaction rates reaching 0.5005 mmol·L^−1^·min^−1^, approximately 8.3 times higher than that under control (blank) conditions.^31^

From the perspective of enhancing the SFGDA catalytic oxidation rate, although the aforementioned strategies have demonstrated feasibility in laboratory-scale systems, their industrial-scale implementation still faces numerous challenges, such as the stability and recyclability of catalysts, operational and maintenance costs of auxiliary equipment (e.g., ozone generators and electrode materials), and the selectivity of reaction pathways under complex multi-ionic background conditions. Therefore, future research should be mechanism-driven and focus on the practical need for efficient catalytic oxidation under high-pH conditions, aiming to develop integrated catalytic systems with pH adaptability, low corrosivity, and sustainable operability.

### Interference by chloride ions

In CaSO_3_ catalytic oxidation systems, chloride ions, as typical coexisting anions, can suppress oxidation efficiency through multiple inhibitory mechanisms. According to Lu et al., when the HCl addition is below 10%, the CaSO_3_ oxidation conversion rate may drop to approximately 85% of its original value, corresponding to a maximum inhibition of around 15%. When the HCl addition increases to 10–16%, the inhibition becomes even more pronounced.^77^ The underlying mechanisms can be attributed to several factors. First, Cl^−^ competes with SO_3_^2−^ for adsorption sites on the CaSO_3_ particle surface, preferentially occupying active sites and thereby reducing the effective attachment of oxygen or catalyst species, which in turn lowers the activation efficiency of SO_3_^2−^.^78^ In addition, Cl^−^ can complex with metal catalysts (e.g., Fe^2+^, Mn^2+^) to form inert coordination compounds, thereby disrupting the catalytic cycle of metal ions and exhibiting a pronounced catalyst poisoning effect.^64^ Moreover, at high concentrations, Cl^−^ may interfere with the solution’s pH buffering capacity and radical stability, further exacerbating the instability of the reaction system.^79^

To address the above issues, various strategies for Cl^−^ removal and passivation have been proposed and preliminarily validated in related wastewater treatment applications. First, the Friedel’s salt precipitation method can immobilize Cl^−^ into the solid phase via a cation exchange mechanism, enabling relatively thorough Cl^−^ removal during pretreatment stages, and is suitable for small-scale, batch-type treatment processes.^80^ Electrodeionization (EDI) is a continuous operation technology based on electric fields and ion exchange membranes, which enables the dynamic removal of Cl^−^ without interrupting the ongoing reaction system, making it suitable for highly automated industrial reaction systems.^81^ Adsorption methods using layered double hydroxides (LDHs), such as ZnMgAl-LDHs, have shown strong selectivity toward Cl^−^, with adsorption efficiencies reaching up to 92%, indicating promising engineering applicability.^82^

Although the above technologies have demonstrated effectiveness in laboratory or pilot-scale systems, several challenges must be addressed before their integration into CaSO_3_ oxidation systems. These include potential declines in removal efficiency and accumulation effects in continuous operation systems, as well as the influence of highly alkaline and calcium-rich backgrounds on Cl^−^ migration behavior during the reaction. Additional challenges include material costs, membrane fouling, and sludge disposal, which may impose both economic and environmental burdens. Therefore, Cl^−^ management in CaSO_3_ oxidation systems should adopt a combined strategy of source control and in-process regulation, in order to ensure both process controllability and maximized efficiency.

### Exogenous catalytic additives

In the catalytic oxidation of CaSO_3_, external additives enhance reaction efficiency through multiple synergistic pathways, with the main mechanisms including: (1) regulating pH to optimize SO_3_^2−^ speciation and improve CaSO_3_ solubility,^83^^,^^84^ which facilitates catalyst-induced radical generation^85^; (2) complexing with or activating Ca^2+^ ions to enhance their reactivity^86^; (3) modifying the gas–liquid interface to increase O_2_ solubility and improve contact efficiency between reactants.^87^^,^^88^ For example, organic acids such as citric acid and acetic acid, when added at appropriate concentrations, can buffer the solution pH to the optimal oxidation range, and form Ca–acid complexes that increase CaSO_3_ solubility, with experimental results showing that the oxidation rate increased significantly from 8.92% to 98.49% in the presence of ammonium citrate.^89^

Surfactants such as sodium dodecyl sulfate (SDS) can enhance mass transfer rates during CaSO_3_ catalytic oxidation by reducing interfacial tension and increasing the gas–liquid contact area. Studies have shown that at a concentration of 50 mg/L, SDS can increase the O_2_–water mass transfer coefficient by up to 2.3 times, resulting in over 30% improvement in the overall reaction rate.^90^ In addition, metal-doped additives such as oxygen-containing bismuth compounds (BiOx) and yttrium oxide (Y_2_O_3_) can effectively modulate the surface electronic structure of catalysts, thereby improving O_2_ activation and facilitating radical chain propagation. For example, the combination of bismuth oxide with platinum catalysts can form Pt–[O]_X_–Bi cluster structures, leading to a 30–40% enhancement in oxidation activity within the catalytic system.^91^ Composite additive combinations have also demonstrated excellent catalytic synergy, such as the co-addition of citric acid and Na_2_SO_4_, which can increase the SO_4_^2−^ formation rate to 0.091 mmol·L^−1^·s^−1^ while maintaining desulfurization efficiency above 96%.^65^

Although external additives can effectively enhance the oxidation efficiency of CaSO_3_, their practical application still faces several challenges. These include uncertainties regarding the economic feasibility and long-term stability of additive dosing. Additionally, additives may interfere with catalytically active sites or alter oxidation pathways. They may also introduce by-products, induce reverse complexation, or quench reactive radicals.^92^^,^^93^ Therefore, in industrial applications, the selection of additives should be guided by the interplay between CaSO_3_ oxidation kinetics and mass transfer conditions, in order to identify additive combinations that are highly compatible with the catalytic system, and to progressively develop precise and controllable multi-additive modulation models to maximize their synergistic enhancement potential.

### Comprehensive evaluation and process guidance

In summary, the WCO of SFGDA faces four key bottlenecks: mass-transfer limitations, pH mismatch, Cl^−^ interference, and inadequate co-additive synergy. At the lab scale, nanobubble technology can rapidly probe kinetic limits, whereas scale-up is better served by energy-efficient micro-reactors or ultrasound-bubble coupling. Radical-chain reactions are favored in mildly acidic media; under strong alkaline conditions, the process relies on O_3_, electrochemical routes, or surface-defect pathways, while the mechanism at neutral pH remains unexplored. Mitigation of Cl^−^ inhibition should be stratified by treatment scale: precipitation for small systems, LDH adsorption for medium systems, and dynamic de-chlorination via EDI for large-scale applications. The selection of additives should balance buffering, complexation, and mass-transfer enhancement; the “citric acid + SDS” combination currently offers the broadest applicability. Overall, only a holistic optimization of mass-transfer intensification, pH alignment, low-chloride operation, and multi-additive synergy can provide a sustainable technological foundation for the industrial scale-up of SFGDA catalytic oxidation.

## Recent advances in catalyst research

**Figure 8:**
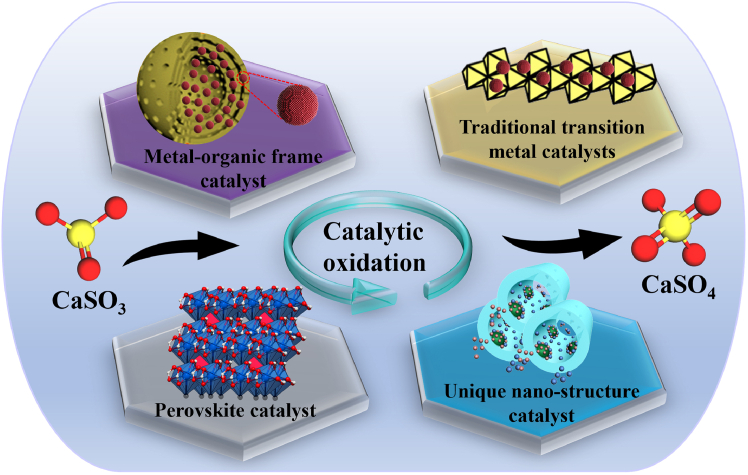
New approaches to catalyst design

Current research on the WCO of CaSO_3_ primarily focuses on four categories of catalysts (as depicted in Figure 8): including the well-established traditional transition metal catalysts, as well as emerging MOFs, perovskite-based catalysts, and catalysts with specialized nanostructures (see Table 2). These catalyst types exhibit distinct advantages and challenges in terms of active site construction, oxidation mechanisms, and environmental adaptability, forming the core technical roadmap of current research.

**Table 2:** Summary of materials for sulfite catalytic oxidation

Type	Catalyst	Reported active sites	Catalytic conditions	Catalytic oxidation performance	Major challenge
Traditional transition metal catalysts	CoSO_4_^94^	Co(II)	pH 10.4; 20°C–45°C; O_2_ 0.21–1atm	activation energy; E = 41.3 kJ/mol	mass transfer, high pH, catalyst cost
	CuCl^95^	Cu(I), Fe(II), Mn(II)	Cu(I) 16 μmol/L; pH 2–4; 45 °C; Air 1.39 × 10^−4^ m^3^/s	Fe(II) > Mn(II) > Cu(I); 0.0034 mmol·L^−1^ s^−1^	solubility of catalyst, low pH conditions
	SA-Fe/Co-CM^96^	Co(II), Co(III), Fe(III)	25 °C; air 400 mL/min	0.01774 mmol·L^−1^ s^−1^	reunion blocking, catalyst cost, mass transfer stability
	Co/CNTs^97^	Co(II), Co(III)	0.5 g/L catalyst; pH 8; 45 °C; air 60 L/h	0.069 mmol·L^−1^ s^−1^; E = 23.43 kJ/mol	mass transfer, reunion blocking, catalyst cost
	Co@NC^98^	Co(II), Co(III)	0.5 g/L catalyst; pH 8; 45 °C; air 60 L/h	0.056 mmol·L^−1^ s^−1^; E = 1.13 kJ/mol	mass transfer, high pH, solubility of catalyst
Metal-organic frame catalyst	Co-MOF (BTC)^56^	Co(II), Co(III)	0.5 g/L catalyst; pH 8; 45 °C; air 60 L/h	0.112 mmol·L^−1^ s^−1^; E = 26.82 kJ/mol	mass transfer, high pH, catalyst cost
	Cu/Fe bi-MOFs^99^	Cu(II), Fe(III)	0.1 g/L catalyst; pH 8; 25 °C; air 60 L/h	0.133 mmol·L^−1^ s^−1^	anions (Cl^−^, HCO_3_^−^, and so forth) inhibition
	Fe/Mn-MOFs^100^	Fe(II), Fe(III), Mn(III), Mn(IV)	2 g/L catalyst; pH 4; 45 °C; air 30 L/h;	3 h CaSO_3_→CaSO_4_; 96.09%	mass transfer, low pH conditions, solubility of catalyst
	Fe/Mn(BDC)(DMF, F)^101^	Fe(III), Mn(III), Mn(IV)	2 g/L catalyst; pH 4; 45 °C; air 12 L/h	3 h CaSO_3_→CaSO_4_ 90.71%; 0.042 mmol·L^−1^ s^−1^	mass transfer, low pH conditions
Perovskite catalyst	MnTiO_3_^102^	Mn(II), Mn(III)	1 g/L catalyst; pH 8; 45 °C; air 60 L/h	0.074 mmol·L^−1^ s^−1^	mass transfer, pH control
	BiCo_0__.__5_Cu_0__.__5_O_3_-MnO_2_^103^	Co(III), Cu(II), Mn(IV)	1 g/L catalyst; pH 6.8; 25 °C; air 60 L/h	0.065 mmol·L^−1^ s^−1^	mass transfer, catalytic additives, solubility of catalyst
	PCaCu_3_Ti_4_O_12_(CCTO)^104^	Cu(I), Cu(II), Ti(III), Ti(IV)	0.05 g/L catalyst; pH 8; 25 °C; air 60 L/h	0.022 mmol·L^−1^ s^−1^	mass transfer, anions inhibition, pH control
	LaCeO_3_^105^	Ce(III), Ce(IV)	0.3 g/L catalyst; pH 5–7; 25 °C; air 60 L/h	0.0729 mmol·L^−1^ s^−1^	mass transfer, anions inhibition, pH control
	ZnCe_0__.__4_Fe_1__.__6_O_4_^106^	Zn(II), Zn(III), Fe(II), Fe(III), Ce(III), Ce(IV)	0.1 g/L catalyst; pH 9; 25 °C; air 60 L/h	0.086 mmol·L^−1^ s^−1^	mass transfer, anions inhibition, catalyst cost, catalytic additives
Unique nanostructured catalyst	MnO_2_@Al_2_O_3_^31^	Mn(III), Mn(IV)	0.05 g/L catalyst; pH 9; 25 °C; air 24 L/h	0.083 mmol·L^−1^ s^−1^; E = 1.89 kJ/mol	mass transfer, suitable alkalinity, catalyst cost
	ZnxCu1-xFe_2_O_4_^107^	Fe(II), Fe(III), Cu(III), Cu(II)	0.2 g/L catalyst; pH 7; 25 °C; air 60 L/h	0.063 mmol·L^−1^ s^−1^	mass transfer, pH control
	CoFeW-LDHs^108^	Fe(II), Fe(III), Co(II), Co(III),	0.2 g/L catalyst; pH 7; 25 °C; air 60 L/h	0.05 mmol·L^−1^ s^−1^	catalytic additives, low pH conditions, catalyst cost

According to current studies on sulfite catalytic oxidation, traditional transition metal catalysts are mainly based on Co, Fe, Cu, and Mn compounds.^41^^,^^94^^,^^95^^,^^109^^,^^110^^,^^111^ Building on this foundation, recent advances have led to the development of three major branches: metal–organic framework (MOF) catalysts, perovskite-based catalysts, and catalysts with unique nanostructures.^57^^,^^112^^,^^113^ This review summarizes representative catalytic materials from each of these categories, with details on their structural composition, active sites, and radical-generation mechanisms listed in Table 1.

### Traditional transition metal catalysts

Traditional transition metal catalysts mainly focus on Co, Fe, Cu, Mn, and their respective compounds. Among them, cobalt- and manganese-based compounds exhibit particularly significant catalytic performance in sulfite oxidation. Experiments indicate that manganese sulfate (MnSO_4_) exhibits superior catalytic activity toward CaSO_3_, with an oxidation rate of up to 0.01 mmol·L^−1^·s^−1^ at an Mn^2+^ concentration of 4.41 × 10^−2^ mol/L, mainly due to the formation of active centers via the substitution of Ca^2+^ by Mn^2+^.^111^ Other studies reported that with the addition of cobalt sulfate (CoSO_4_) at 45 °C, the oxidation rate of SO_3_^2−^ reached 0.06 mmol·L^−1^·s^−1^, significantly higher than the uncatalyzed condition, attributed to the stable redox cycling of Co^2+^ ions within the lattice, which promotes the oxidation process. Iron ions show moderate catalytic activity toward CaSO_3_ oxidation, but their performance is generally lower than that of Co and Mn. In experiments by Karatza et al., Cu^+^-catalyzed SO_3_^2−^ oxidation exhibited a rate only 1.5 times higher than the uncatalyzed condition, with significantly lower activity compared to Co- and Mn-based catalysts, indicating limited effectiveness of Cu^+^ alone.^95^ In addition, Li et al. investigated the catalytic performance of hydrophobic membranes loaded with Fe–Co bimetallic oxides in sulfite oxidation. The combination of Fe_2_O_3_ and Co_3_O_4_ significantly enhanced the oxidation rate—by a factor of 5.8—with only a 15% decline in catalytic activity after seven cycles.^96^ For the oxidation of magnesium sulfite in Mg-based desulfurization systems, Li et al. prepared Co/CNTs catalysts by loading CoO and Co_2_O_3_ onto multi-walled carbon nanotubes. At a cobalt loading of 30%, the apparent activation energy of the oxidation reaction was 23.43 kJ/mol. The overall reaction rate was controlled by internal oxygen diffusion, and the catalyst played a critical role in enhancing O_2_ mass transfer in the three-phase system.^97^ Liu et al. developed a Co–Mn/AC (activated carbon from biomass) catalyst to enhance magnesium sulfite oxidation in Mg-based desulfurization systems. Experiments indicated that the incorporation of Mn_3_O_4_ and MnO_2_ improved the dispersion of active sites, and the Co^2+^/Co^3+^ redox cycling significantly enhanced the oxidation rate, reaching up to 0.056 mmol·L^−1^·s^-1^.^98^

To further improve oxidation efficiency, recent research has increasingly shifted toward new types of catalysts: MOF catalysts, perovskite-based catalysts, and catalysts with unique nanostructures. MOFs significantly enhance catalytic activity through their highly tunable pore structures and multifunctional active sites, while perovskite catalysts exhibit excellent redox properties and thermal stability, making them highly effective in wet flue gas desulfurization processes. Nanostructured catalysts further improve oxidation performance by offering larger specific surface areas and enhanced synergistic effects. These materials leverage properties such as structural tunability and multivalent redox cycling to significantly improve both the efficiency and selectivity of sulfite oxidation reactions.

### MOF catalyst

MOF catalysts are a class of highly ordered porous materials formed by coordination bonds between metal centers and organic ligands.^114^ Due to their unique structural features and high specific surface areas, MOF catalysts exhibit notable advantages in catalytic reactions, including high activity, selectivity, tunability, and regenerability.^115^ In the context of magnesium-based desulfurization, cobalt-based MOFs (Co-MOFs) have been applied to catalyze the oxidation of MgSO_3_. Geng et al. demonstrated that a Co-MOF (BTC) catalyst synthesized via a one-step ultrasound-assisted method exhibited excellent dispersion and catalytic activity. The oxidation rate catalyzed by Co-MOF was 13–14 times higher than that under non-catalytic conditions, reaching 0.112 mmol·L^−1^·s^−1^, and 4–5 times higher than that of Co-SBA-15. The apparent activation energy was 26.82 kJ/mol, and the catalyst exhibited excellent stability and reusability.^56^ Zhao et al. used MOF-templated CuFe_2_O_4_ to activate sulfite auto-oxidation and simultaneously remove organic pollutants. Under mildly alkaline conditions (pH = 8.0), the CuFe_2_O_4_ catalyst significantly enhanced the sulfite oxidation rate, enabling over 80% pollutant removal within 2 min. The redox cycling between Cu(II) and Fe(III) was found to be critical for sulfite auto-oxidation.^99^ Su et al. developed a Fe/Mn bimetallic MOF catalyst with defect structures induced by carboxylic acids to enhance the catalytic oxidation of CaSO_3_ in desulfurization ash. The oxidation rate of CaSO_3_ increased from 2.76% to 96.09% within 3 h, with a peak rate of 0.078 mmol·L^−1^·s^−1^, and only a 10.42% decrease in catalytic activity after five reuse cycles. The defect structures promoted the generation of O_2_·−, thereby accelerating the oxidation reaction.^100^ Su et al. also synthesized a Fe/Mn-BDC (DMF, F) bimetallic MOF catalyst, which exhibited excellent performance in CaSO_3_ oxidation. Experimental results showed that the oxidation efficiency of CaSO_3_ reached 90.71%, with an oxidation rate of 0.042 mmol·L^−1^·s^−1^, significantly outperforming single-metal catalysts.^101^

The aforementioned MOF-based catalysts, with their unique structural advantages and high catalytic performance, demonstrate great application potential in the catalytic oxidation of CaSO_3_. In particular, their excellent reusability and stability make them ideal candidates for catalytic reactions.^28^^,^^29^ However, in practical applications for SFGDA oxidation, MOF catalysts may suffer from fouling-induced deactivation.^116^ Calcium salts generated from SFGDA and during the oxidation process may react with MOF pore channels, leading to the formation of deposits that clog the pores and reduce the effective surface area and catalytic activity.^117^ Catalytic additives can enhance the solubility of reactants and reduce calcium salt precipitation.^22^ In addition, the use of chelating agents such as EDTA or citric acid can complex calcium ions, thereby lowering the likelihood of salt deposition and mitigating fouling issues.^118^^,^^119^ Furthermore, strategies such as metal center modification (e.g., incorporating more stable metal ions such as Ti^4+^ and Mo^6+^),^120^ surface functionalization (e.g., fluorination or silanization),^121^ and composite material design (e.g., combining with inorganic or polymeric matrices) can improve the anti-fouling and anti-pollution properties of MOF catalysts.^122^^,^^123^^,^^124^ Meanwhile, regeneration and recovery techniques for MOF catalysts are also continuously being developed, including thermal treatment or acid–base washing for activity recovery, and the development of magnetic MOFs for rapid separation and reuse.^125^^,^^126^^,^^127^ Through multidimensional regulation, these issues of pore blockage and fouling-induced deactivation in the WCO of SFGDA can be effectively alleviated, thereby enhancing both catalytic efficiency and operational lifespan.

### Perovskite catalyst

Perovskite-based catalysts are a class of composite oxide catalysts with a typical ABO_3_-type structure, widely used in various catalytic reactions.^128^ Their unique crystal structures and tunable compositions confer high activity, selectivity, and stability in environmental remediation, energy conversion, and industrial chemical processes.^129^ Qi et al. synthesized a stable and efficient perovskite catalyst, MnTiO_3_, which effectively promoted the oxidation of CaSO_3_. Compared to non-catalyzed conditions, the oxidation rate of CaSO_3_ increased by 4.3 times to 0.074 mmol·L^−1^·s^−1^, and the yield of CaSO_4_ increased by 34.02%. After four reuse cycles, the oxidation efficiency declined by only 9.57%, indicating good durability and low manganese leaching.^102^ Gu et al. synthesized an efficient BiCo_0.5_Cu_0.5_O_3_–MnO_2_ catalyst via a hydrothermal method, featuring a larger specific surface area and more uniform pore structure, which prevents perovskite agglomeration. This composite catalyst was shown to effectively activate CaSO_3_ and generate radicals such as SO_4_·− and SO_5_·−. Moreover, the valence state transitions of Co, Cu, and Mn were identified as the main contributors to CaSO_3_ catalytic oxidation, demonstrating strong synergistic effects and playing a key role in the catalytic process.^103^ CaCu_3_Ti_4_O_12_ is a new perovskite catalyst that has shown remarkable performance in CaSO_3_ oxidation and arsenic [As(III)] removal. Under experimental conditions of 0.05 g/L CaCu_3_Ti_4_O_12_ and 0.4 mmol/L sulfite, Shao et al. achieved a maximum CaSO_3_ oxidation rate of 0.022 mmol·L^−1^·s^−1^ within 30 min, accompanied by simultaneous As(III) removal. The catalyst promoted the formation of SO_4_·−, thereby accelerating the oxidation of CaSO_3_. Furthermore, CaCu_3_Ti_4_O_12_ exhibited high reusability and structural stability during the reaction process.^104^ Meng et al. synthesized a LaCeO_3_ perovskite catalyst using a citric acid sol–gel method, which demonstrated excellent stability in the activation of bisulfite oxidation. During the catalytic process, SO_4_·− and ·OH radicals were generated. Characterization indicated that the reaction was initiated by *in situ* surface regeneration on the catalyst. The Ce^3+^/Ce^4+^ redox cycle was identified as the primary factor promoting oxidation.^105^ Qin et al. prepared an oxygen vacancy-rich perovskite catalyst (ZnCe_0.4_Fe_1.6_O_4_), which achieved efficient sulfite activation and pollutant degradation via enriched oxygen vacancies and multivalent redox cycles of Zn, Ce, and Fe. In the ZnCe_0.4_Fe_1.6_O_4_/sulfite system, SO_4_·− and ·OH were identified as the dominant ROS. The redox cycles of Zn^2+^/Zn^3+^, Fe^2+^/Fe^3+^, and Ce^3+^/Ce^4+^ were critical to sulfite activation, with surface oxygen vacancies further enhancing the activation process.^106^

Perovskite catalysts have demonstrated excellent performance in SO_3_^2−^ oxidation, owing to their unique multivalent redox cycling, oxygen vacancy structures, and radical-generation mechanisms, resulting in highly efficient and stable oxidation behavior.^30^^,^^130^^,^^131^ However, most perovskite catalysts are in powder form, which poses challenges for recovery and reuse, and limits their application to homogeneous catalysis.^132^^,^^133^ Converting perovskite catalysts into supported forms or composites with other materials allows the transformation from homogeneous to heterogeneous catalysis, which not only improves recoverability but may also enhance catalytic performance.^130^ For instance, Gu et al. prepared a composite of perovskite and MnO_2_, which optimized the pore structure and effectively prevented perovskite agglomeration.^103^ Another approach involves combining perovskite catalysts with metallic frameworks to fabricate supported catalytic materials.^134^^,^^135^ Such integration with metallic substrates can reduce metal leaching and improve reaction stability.

### Unique nanostructured catalyst

Nanostructured catalysts are a class of catalysts whose catalytic performance is enhanced through deliberate nanoscale structural design. These nanostructures not only increase the surface area and number of active sites but also influence electron transfer, mass transport pathways, and reaction selectivity, thereby significantly improving catalytic efficiency.^136^^,^^137^ Meng et al. developed a MnO_2_-functionalized hydrophobic ceramic membrane by loading sea urchin-shaped β-MnO_2_ nanoparticles. This catalyst significantly enhanced the oxidation rate of sulfite, particularly under mildly alkaline conditions. Experimental results showed that the MnO_2_ membranes loaded with 1.6–4.5 mg exhibited 5.4 to 8.3 times higher catalytic efficiency for Na_2_SO_3_ oxidation than the non-catalyzed system, with a maximum oxidation rate of 0.074 mmol·L^−1^·s^-1^.^31^ In another study, Huang et al. synthesized magnetic ZnxCu_1-X_Fe_2_O_4_ nanomaterials using a sol–gel combustion method. Under UV–visible light irradiation, the catalyst exhibited excellent photocatalytic performance for sulfite activation and pollutant degradation, maintaining high activity even after four consecutive cycles. The generation of SO_4_·− radicals facilitated by surface Fe(III) and Cu(II) species was identified as the dominant mechanism.^107^ Layered double hydroxide (LDH) nanomaterials have also shown promise for CaSO_3_ catalytic oxidation due to their unique layered structures and tunable composition. The abundant surface active sites and good ionic conductivity of LDHs contribute to enhanced oxidation efficiency.^32^^,^^138^ By tuning the metal ion composition, LDH-based catalysts can be engineered to optimize active site distribution, improve selectivity, and accelerate reaction kinetics.^139^ Chen et al. developed a CoFeW-LDHs catalyst for sulfite activation and pollutant degradation. This material featured vertically aligned ultrathin nanosheets forming a porous network, which enhanced CaSO_3_ activation. Tungsten doping promoted redox cycling between Co(II)/Co(III) and Fe(III)/Fe(II) on the catalyst surface, facilitating the sustained production of ROS such as O_2_·− and SO_4_·−. The LDH-based catalyst also maintained high performance over multiple reuse cycles. For instance, a Pd-based LDH catalyst demonstrated outstanding stability in hydrogenation reactions, maintaining over 90% catalytic activity after 20 cycles. This provides a new direction for the design of durable catalysts for CaSO_3_ oxidation.^108^

Nanostructured catalysts have shown remarkable potential in the catalytic oxidation of CaSO_3_, particularly in improving catalytic efficiency and reaction selectivity. Strategies such as designing sea urchin-like nanostructures, layered double hydroxides (LDHs), and metal-doped materials have contributed to enlarged specific surface areas, enriched active sites, and enhanced electron transfer processes.^140^^,^^141^ However, nanomaterials tend to suffer from agglomeration, which leads to reduced catalytic activity. Moreover, issues related to catalyst recovery remain unresolved.^142^ Recent studies have proposed the design of core–shell structured catalysts to improve durability and stability.^143^ One approach involves coating metal oxides (e.g., Fe_3_O_4_ or TiO_2_) over catalytically active metals such as platinum, gold, or silver to form metal-core/oxide-shell architectures. These structures enhance the oxidation resistance and durability of the catalysts.^144^ Such core–shell configurations can effectively reduce metal leaching and improve both catalyst stability and recoverability, exhibiting strong resistance to deactivation in oxidation reactions.^145^ Applying this strategy to nanostructured catalysts offers a promising route to overcome their inherent deactivation issues and improve long-term catalytic performance.

Overall, bimetallic MOF catalysts leverage tailorable pores and multivalent redox cycles to accelerate oxidation via O_2_·^-^. Perovskite catalysts generate SO_4_·^-^ and SO_5_·^-^ radicals via metal-valence cycling and oxygen vacancies, featuring low metal leaching and excellent recyclability. Nanostructured catalysts, endowed with high surface area and layered/pore-facilitated mass transfer, boost oxidation in mildly alkaline media; membrane immobilization or magnetic core–shell designs preserve reusability, although particle agglomeration must be controlled. In comparison, MOFs deliver the fastest kinetics, perovskites suit complex aqueous chemistries, and nanostructured catalysts show the greatest promise under alkaline conditions.

**Figure 9:**
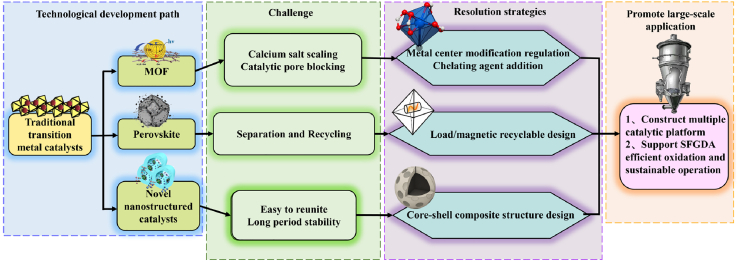
The technological development path of catalysts and the challenge of industrialization

However, the high catalytic activity observed at the laboratory scale does not necessarily translate into reliable industrial performance (see Figure 9). During scale-up, the three new catalyst types still face distinct bottlenecks: MOF catalysts are prone to Ca-salt deposition that blocks pores and deactivates the structure^115^; powdered perovskite catalysts suffer from poor separation and recycling efficiency^30^; and nanostructured catalysts tend to aggregate and lack durability.^142^ Several engineering approaches can mitigate these limitations: metal-center modification^29^ and chelating-agent antiscaling^89^ to enhance fouling resistance, along with support/magnetic recovery designs and core–shell composites^143^^,^^145^ to improve recyclability and stability. Collectively, these measures could establish a versatile catalytic platform that enables efficient and sustainable SFGDA oxidation, laying a solid foundation for its large-scale implementation.

### Comprehensive comparison and design guidelines

MOF catalysts can increase the sulfite oxidation rate by more than an order of magnitude,^56^ yet are susceptible to pore blockage by Ca-salt deposition. Perovskite catalysts deliver efficient and stable oxidation through multivalent redox cycles with minimal metal leaching; however, powdered forms are difficult to separate and recycle. Nanostructured catalysts, with their enormous surface area, accelerate oxidation 5- to 8-fold in mildly alkaline media,^31^ but suffer from aggregation and limited durability. Conventional transition-metal catalysts are mature and easy to operate, yet offer only modest performance gains. Catalyst selection should therefore balance target rate, process conditions, and ease of recovery. When a very high rate is required and scaling can be mitigated with chelators or MOF modifications, MOF catalysts are preferred. If stability and low metal release are paramount, supported perovskite catalysts are advisable for repeated use. Under mildly alkaline conditions or when membrane/particle integration is desired, nanostructured catalysts are suitable, but their anti-deactivation must be enhanced via core–shell design. Conventional catalysts remain a low-cost, readily available backup for applications with modest performance requirements. Strategies such as metal-center doping, support immobilization, chelator antiscaling, and core–shell composites can simultaneously enhance anti-scaling, recyclability, and stability, underpinning efficient and sustainable SFGDA oxidation.

## Conclusion and prospect

The WCO of SFGDA offers an efficient pathway for the stabilization and resource utilization of CaSO_3_. Through a free-radical chain reaction mechanism and catalyst site-occupancy interactions, this process enables the rapid conversion of CaSO_3_ to CaSO_4_ under mild conditions, producing stable products suitable for construction material applications. This review systematically evaluates mechanisms, rate-limiting factors, and catalytic systems, yielding three key consensus points.

(1) Catalyst performance ranking: in weakly acidic to neutral media, MOF catalysts deliver the fastest kinetics via tunable pores and multivalent redox cycles; in alkaline SFGDA slurries, supported perovskites combine high rate with structural stability; nanostructured or core–shell catalysts, offering large surface area and easy recovery, are cost-effective options under alkaline conditions.
(2) Mechanistic essentials: radical-chain reactions (SO_3_·−, SO_4_·−, SO_5_·−) combined with lattice occupation–defect synergy drive efficient oxidation, while their activation efficiency is strongly coupled to mass transfer, pH, and Cl^−^ levels.
(3) Engineering hurdles: in highly alkaline or high-Cl^-^ environments, catalyst deactivation, poor O_2_ mass transfer, and scaling constitute the principal limitations during scale-up.

Based on this review of mechanisms, rate-limiting factors, and catalytic systems, future research should prioritize the following four directions.

(1) Precise catalyst design and deactivation mitigation. In weakly acidic to neutral media, MOF-derived catalysts with tunable pores and multivalent redox cycles deliver the fastest kinetics; in alkaline SFGDA slurries, supported perovskites combine high reaction rates with structural stability; nanostructured or core–shell composites, offering high surface area and easy separation, provide an economical alternative. Future work should explore bi-/multimetal defect engineering (e.g., Fe–Mn, Co–Ce) to enhance radical generation and chloride tolerance. Density-functional theory (DFT) calculations should be employed to clarify valence cycling and occupation–defect coupling at active centers, thereby guiding quantitatively controlled catalyst design.
(2) Process intensification under alkaline or high-chloride conditions. Elevated pH and high chloride levels are the principal barriers to efficient radical-chain reactions. Alkali- and chloride-resistant catalysts, or the introduction of pH buffers and complexing microenvironments, should be developed to cut acid consumption while suppressing scaling. Integrating nanobubbles, micro-interface reactors, and ultrasound/electrochemical oxidation can boost O_2_ mass transfer and radical generation rates. For Cl^−^-rich SFGDA, precise de-chlorination should be conducted before reaction via Friedel’s salt precipitation or EDI.
(3) Scale-up of recyclable catalysts and scaling control. Industrial scale-up must first address catalyst recovery and system scaling. Magnetic core–shell or porous-supported catalysts enable rapid separation and reuse, while external complexing agents can slow scale formation and ensure continuous stable operation.
(4) Environmental and economic assessment. To validate the industrial sustainability and economics of the wet catalytic oxidation of SFGDA, a full life cycle quantification is essential. The assessment should include catalyst synthesis, operation, regeneration, and final disposal, with emphasis on energy use, greenhouse-gas emissions, and chloride migration risk. Cl^−^-containing effluents should be managed via zero-discharge or resource-recovery schemes to prevent secondary pollution. Economic analysis should compute unit processing costs and evaluate gypsum by-product revenue in comparison with current landfilling or stockpiling practices. If the by-product gypsum has commercial value and processing costs fall below those of landfilling, the technology’s adoption potential will rise markedly.

With ongoing improvements in catalyst performance and process optimization, WCO of SFGDA is expected to achieve higher oxidation rates and lower operating costs, thereby playing a greater role in solid-waste valorization and offering a practical solution for the large-scale utilization of desulfurization residues.

## Data availability

Data sharing is not applicable.

## Acknowledgments

We acknowledge financial support from the Special Fund for Talent Development in 2024 Led by Yunnan Provincial Department of Human Resources and Social Security (Grant No. 53000021110000107950), the Key R&D Plan Projects in Yunnan Province (Grant No. 202203AC100002-03), the Yunnan Province Agricultural Basic Research Joint Special Project (Grant No. 202301BD070001-065) and the Scientific Research Fund of Yunnan Provincial Education Department (Grant No. 2022J0524).

## Author contributions

Writing—review and editing, H.-Q.X. and J.-Z.Q.; conceptualization, Z.-X. L.; funding acquisition, review and editing, X.-L.S and X.-Y.M. All authors have read and agreed to the published version of the article.

## Declaration of interests

The authors declare no conflicts of interest.

## Supplemental information

Table S1. Literature matrix includes catalyst types, mechanistic pathways and limiting factors Notes: ○ = Main catalytic oxidation mechanism, ✓ = Catalytic control factor, Blank= Not explicitly mention.
